# Supplementary material for: Content-rich biological network constructed by mining PubMed abstracts
Source: BMC Bioinformatics. 2004 Oct 8;5:147. doi: 10.1186/1471-2105-5-147 (PMC528731; doi:10.1186/1471-2105-5-147)
Supplement: Additional File 2 — The original results of the above study (non-essential files are deleted to keep the file size under the limit set by BMC bioinformatics). [file 1471-2105-5-147-S2.bz2 › chilibotAdditionalFile2/dip05/307ID9117348E1305/html/CD9_SDC1.html]

 


 **CD9** and **SDC1** 
  
Found 5 abstracts in PubMed, retrieved 05.  
 

 What does Google say? 
 PDF only 
| .edu only 

---

**Interactive relationship** (e.g. stimulation, inhibition, etc)

**Neutral relationship**- However, after prolonged culture of FO B cells with LPS, surface  **CD9**  was induced, together with syndecan 1  [ **SDC1** ] , indicative of plasma cell differentiation.  Ref: 12023357 J Immunol, 2002
- **CD9**  was detected in beta 1 integrin immunoprecipitates and also in immunoprecipitates of CD44 and syndecan  [ **SDC1** ] , but not of cadherins.  Ref: 9117348 Cell Adhes Commun, 1996
- Immunophenotypic expression was similar for CD38, CD138  [ **SDC1** ] , CD2, CD3, CD16, CD10, CD13, and CD15, but PCL differed from MM in the expression of CD56,  **CD9**  HLA DR, CD117, and CD20 antigens.  Ref: 9920853 Blood, 1999
- Three patterns of antigen expression were detected 1 markers constantly positive in all cases analyzed  **CD9** , CD29, CD33, CD43, CD44, CD49d, CD49e, CD51, CD71, CD117, and Fc epsilon RI, 2 antigens that were constantly negative CD1a, CD2, CD3, CD5, CD6, CD11a, CD14, CD15, CD16, CD19, CD20, CD21, CD23, CD25, CD30, CD34, CD38, CD41a, CD42b, CD65, CD66b, HLA DR, and CD138  [ **SDC1** ] , and 3 markers that were positive in a variable proportion of cases CD11b 50%, CD11c 77%, CD13 40%, CD18 20%, CD22 68%, CD35 27%, CD40 67%, CD54 88% and CD61 40%.  Ref: 9699944 Anal Cell Pathol, 1998
- Sodium deoxycholate SDC  [ **SDC1** ]  and octyl beta glucoside OG, structurally similar to CHAPS and Brij35 and disrupting the lipid bilayer, showed a modest increase of MRP1  [ **CD9** ]  photolabeling with IAARh123.  Ref: 12684054 Biochem Biophys Res Commun, 2003

**Non-interactive relationship** (e.g. studied together, co-existance, homology, etc.)

- Proteolytic digestion of IAARh123 photolabeled MRP1  [ **CD9** ]  labeled in the presence or absence of various detergents CHAPS, SDC  [ **SDC1** ] , or OG revealed identical photolabeled peptides.  Ref: 12684054 Biochem Biophys Res Commun, 2003
